# The Ideology of Creativity and Challenges of Participation

**DOI:** 10.5964/ejop.v11i3.1032

**Published:** 2015-08-20

**Authors:** Michael Hanchett Hanson

**Affiliations:** a Teachers College, Columbia University, New York, NY, USA

Anna Craft, a leading researcher on creativity and education, described a condition that many of us have watched accelerate: “Creativity is an important element of the zeitgeist in the early twenty-first century, worldwide” (2005, p. ix), encompassing discourses on genius and individualism, democracy and politics, the social good, technological advances and educational practice, among others (Banaji, Burn, & Buckingham, 2006 as cited in Craft, 2010). It is important to note just how distinctive our times are in this regard. The ancient world did not subscribe to a psychological view of creativity, and during the Middle Ages in Europe in most places to assert that someone was creative would have been blasphemous (Weiner, 2000). God created. People only made things. In other words, we do not need a psychological concept of creativity to write great literature, develop philosophies, lay the foundations of democracy or build beautiful temples and cathedrals. In addition, the idea of “creating” or being “creative” retained implications of the dangers as well as the promises that come with change until the late nineteenth century. Then a wholly positive view of creativity largely eclipsed its negative connotations as dangerous, hubristic and potentially destructive (Mason, 2003).

Since creativity came to be viewed as almost wholly positive, its importance has steadily grown. In particular, an ever-broader range of creativity theories in psychology and sociology have contributed to the creativity zeitgeist. There is a consensus definition of creativity in social science: producing something novel and of value in a context. As it turns out, though, that definition is just a starting point for a wide range of controversies. Early psychological views of creativity included sublimated infantile desires (Freud’s views of the sources of creative ideas and motivations), sudden restructuring of perception (Gestalt views of insight) and the traits of divergent thinking (ideational flexibility, fluency and originality). Then the humanistic psychologists argued that creativity was the expression of a universal self-actualizing drive and its development was necessary to be a “fully functioning person” (Rogers, 1969, p. 278). Since the cognitive revolution in the mid-twentieth century, psychologists have developed a range of cognitive views from creativity as systemic evolution of thought (Gruber & Wallace, 1999; Wallace & Gruber, 1989) to creativity as decision making (Sternberg, 2003; Sternberg & Lubart, 1991) to creativity as a cognitive variation-selection process (Simonton, 1999). Meanwhile, sociocultural theorists have gone beyond the individual to locate creativity within the dynamics of the social and material worlds (Csikszentmihalyi, 1997, 1999; Glăveanu, 2014b; Sawyer, 2010).

This Cambrian explosion of creativity theories has contributed to a conceptually rich and socially powerful concept within culture. At the same time, the proliferation of theories put the very social scientists who developed them in an increasingly fragmented field without a clearly defined subject of interest. These theorists regularly decry the fact that we do not have a clear view of what we are studying. As another leading creativity researcher, Teresa Amabile, and her associates described well: “Creativity researchers are often accused of not knowing what they are talking about” (Amabile et al., 1996, p. 19).

## Problem or Strength?

After 12 years of teaching theories of creativity to educational practitioners I have come to see the definitional ambiguity of creativity differently than many of my fellow psychologists (Hanchett Hanson, 2013a, in press). I propose that the intransigent problem of definition is actually a strength of the concept of creativity as a cultural phenomenon. Furthermore, all of the social science haggling over definitions and locations of creativity is key to that strength. Our distinctive concept of creativity as it has developed since the nineteenth century has provided a site for debates that are at the center of modern and postmodern society, such as what is an individual and what is the individual’s relation to culture? To what extent do people have agency? What are the sources and nature of change?

Of course, the concept of creativity has not just been a slippery issue for researchers, nor just a site of debate. It reflects a view of the world. Although the various theories are contradictory in many ways, they share fundamental assumptions. For creativity to be a driving force of civilization, crucial to economic growth and personal development, the change that creativity brings must be real and important. At the end of the day, the world cannot be as it always was with core underlying truths to which the ephemera of change are simply distracting. This is not a Platonic world. Neither can the course of individual and social change be entirely predetermined. Human agency must be important. Finally, for creativity to carry the far-reaching value that its rhetoric claims, the value of creative output – new ideas, products, practices and our very selves – must be relatively simple and stable. At least in theory, there must be *one value* for a change, or a predominant value. The complexities of perspectival valuation, of silver linings and unexpected consequences and of shifting cultural views are problematic to the very idea of declaring something definitively “creative” – novel and of value. The various theories of creativity may accommodate complexities of valuation to a point. Overall, though, the complexity ceiling has tended to be fairly low and reliance on institutionalized power structures – eminence – for the final say, high.

In other words, the concept of creativity provides a site to explore important issues within a framework of often unquestioned assumptions. Beyond claims of the specific theories, the amalgam of theories have contributed to an underlying ideology. This ideology is important because it concerns one of the most salient characteristics of our times: change. It is also a fascinating ideology because, in keeping with the values it represents, the ideology changes over time.

## The Challenges of Participation

My view of creativity as ideology is not original. Robert Paul Weiner (2000) wrote about it 15 years ago. Other social and critical theorists have written about creativity as ideology (for example, Raunig, Ray, & Wuggenig, 2011; Rehn & De Cock, 2009). Even one of the most authoritative voices in creativity research, Mark Runco, longtime editor of *Creativity Research Journal,* and Robert Albert, another well-established researcher on the topic, have included ideology as one way to think about creativity (Runco & Albert, 2010).

### Claiming and Taking Responsibility for the Ideology

My own intention in viewing creativity as ideology is not to discredit the concept but to claim it more fully. The challenge is to participate in defining the core concept of this powerful ideology. Every time educators, politicians, businesspeople, philanthropists or social scientists invoke the concept of creativity for their purposes, they participate in the ongoing evolution of the concept. The challenge is to do so knowledgably, intentionally and responsibly. Even if – no, especially if – your participation imposes constraints on change, resisting the neophilic, western-centric, market-driven exuberance of much of the rhetoric of the ideology of creativity, *participate!* Creativity without limits, conceived as the ability to do or be anything, as meaning outside of contexts (“outside the box”), is ideology run amuck. Saying “recreate yourself!” to someone whose life has fallen into chaos after years of work because of economic or technological shifts is insensitive and insulting. The practical necessity may be real and the sense of hope useful. At the same time, the enthusiastic rhetoric celebrates an oppressive slight of hand, individualizing responsibility for systemic problems. Treating traditions that give people’s lives meaning as devalued in the face of “creative” (neophilic) market dynamics can amount to a smokescreen for oppression. And discounting the value of those who devote their lives to maintaining culture – average people as well as scholars and virtuosos – compared to those who change culture is laughable. Without legions of the former the few who get credit for being creative geniuses would have nothing to change. (For different articulations of similar critiques of the ideology of creativity, see Weiner, 2000.)

To be clear, even the most optimistic theories of self-actualization of the humanistic psychologists (for example, Maslow, 1954/1970; Rogers, 1961/1989) did not contend that people could recreate themselves at will – “be anything you want to be.” They all recognized constraints of individual development, and largely aimed at Nietzsche’s imperative: “become what thou art” (1882/1910, p. 209). Yet the rhetorical flourishes that introduce and justify much creativity research imply a panacea for personal, economic and cultural problems.

An alternative to the exaggerated rhetoric of creativity is acceptance of the underlying assumption that change is constant, real and uncertain but necessarily and fortunately occurs within many constraints and social dynamics. From that perspective, individuals, groups and communities have to choose what changes they want to see and what values they want to preserve. They can then advocate well-informed change *within* traditions and can be mindful of the development of themselves and their thought. This is the challenge I present to the educators in my graduate school classes every year. Even though this more restrained view stands in opposition to much of the vaunted rhetoric of creativity, again, it is in line with the actual theories and research. When researchers investigate the lives and thinking of da Vinci, van Gogh or Einstein, they are studying “new ideas” only after the ideas have become canonical. The researchers are studying the evolution of cultural tradition.

### Extending the Ideology to Participation

As noted earlier, I have taught creativity theories for many years, but only recently finished a book putting the views outlined above in the context of the history of psychology. One of the striking themes that I found in writing that book was how consistently creativity theories and research have been used to try to extend the social franchise. From the mid-twentieth century on, the concept of creativity was defined as an alternative to exclusive focus on traditional views of intelligence. Theories of intelligence and intelligence testing have a long history in discourses of racial and cultural dominance (Jensen, 1998; Simonton, 2003). Creativity has been presented as different but equally valuable. J. P. Guilford was already a famous psychometrician when he made his famous 1950 presidential address to the American Psychological Association. Addressing a room dominated by behaviorists and psychometricians, he called for the study of creativity. That speech was just part of a much larger, lifelong goal of disproving the concept of a general intelligence factor (*g-*factor) and replacing it with a wider, more nuanced view of intelligence. Ironically, the view of creativity as a *generalized* trait with divergent thinking as its measure has had enduring impact, while Guilford’s overall view of the structure of intellect has not had the impact he hoped. Emphasis on social inclusion and diversity was not just the interest of psychometricians, though. The humanistic psychologist, Abraham Maslow (1971/1993) was among the most explicit about the ideological goals of creativity. He saw psychology as capable of constructing new kinds of people and specifically wanted to promote “Heraclitian” people (p. 57) who would be extreme individualists and comfortable with constant change. He saw America as the place where such people could best develop, and by promoting the social diversity that came with creativity America would attract people from around to world to its side in the Cold War. In Maslow’s rhetoric, we see the combination of a push for broadening the social franchise, a continued idolizing of the great – “Heraclitian” now replacing nineteenth century “genius” – and an explicit ideology of American cultural domination through psychology. To be sure, the story of the development of the ideology of creativity alongside and through psychology is far from simple or pure. Still, expanding social participation is a deep theme that runs across widely varying theories.

More recently, explicitly participatory models of creativity have come to the fore (Clapp, in press; Glăveanu, 2011, 2014b; Hanchett Hanson, 2013a, in press; Sawyer, 2010; Sawyer & DeZutter, 2009). All of these models retain a concept of individual agency but not individual ownership of ideas. Instead of focusing almost entirely on how to get people to think of new ideas, the participatory models situate ideation within individual development, group dynamics and historical settings. The support roles and the field (gatekeeper) roles that people take up as they *integrate* novelty become more central. Choosing, supporting, interpreting and refining ideas are as important as “having” an idea. Indeed, on close examination, distinctions between field roles and the “creator” role begin to disappear. People we have traditionally called “creators” may be more usefully conceived as “producers,” as Vlad Glăveanu (2014a) has suggested. These are people who champion ideas and organize resources to serve the idea over time. I have noted that the idea of the creative person as “curator” of ideas also applies (Hanchett Hanson, in press). Creator as curator emphasizes the tasks of selecting, emphasizing, and powerfully presenting ideas that – as always – derive from historical domains, broader culture (“commonsense”), the artifacts of culture and other people’s ideas. Freud is an example. His role as a crucial driving force in conceiving, developing and promoting psychoanalysis is obvious. Yet his work took form in the context of a culture where many of “his” ideas were already present, particularly those that had been articulated by Nietzsche. Freud’s particular applications of the ideas came response to patients and discussions with his followers, who were also clinicians. The patients are generally treated as mere objects of his observations, but they were crucial actors in his thought processes. Some ideas from discussions with his followers were integrated into Freud’s writings and presentations, and some he famously rejected (Gay, 1988). (If proponents of rejected ideas did not recant, the person was often ejected from the group along with the idea.) For me, thinking of Freud as a determined and temperamental producer or as a curator trying to find the right, provocative and generative combination of ideas is more helpful than simply thinking of him as a “creator.” The latter term comes with too many magical implications of decontextualized (“outside-the-box”) ideation and god-like *ex-nihilo* conjuring and too few cues of where to look for the psychological or social goals, processes, advantages and costs.

## An Aha! Moment: Education

This all sounds reasonable, I hope. But it may seem a little abstract. My own Aha! moment came when I considered how useful participatory views could be to education. The role of creativity in education has been a persistent concern since at least the mid-twentieth century (for example, Beghetto & Kaufman, 2010; Craft, 2005; Guilford, 1950; Maslow, 1971/1993; Rogers, 1969; Starko, 2014). Like the rest of the creativity discourse, much of the discussion of education has concerned how to be more inclusive of students’ abilities. Trying to promote creativity through education comes with pitfalls, though. For one thing, education traditionally assesses outcomes, and creativity research has provided tests for such assessment, particularly divergent thinking tests, many of which come with age-specific norms. The relation of divergent thinking tests to creative performance is controversial, but the current most optimistic claims are that divergent thinking provides an estimate of a potential for creative work (Runco, 2010). Critics have seen little reason for assuming a causal link between divergent thinking and actual creative performance (for discussions see, Hanchett Hanson, 2013b, in press; Runco, 2010; Weisberg, 2006). There is, then, a validity issue for education – what are we trying to teach, and is this the way to measure it? There is also a strategic question: do we want to designate another education goal by which some students will succeed, many will get by and others will fail? Another way to fail? That may sound excessively curmudgeonly and alarmist, but it brings me to the Aha! moment.

A former student of mine emailed me about her daughter who was a straight-A student and generally liked school. Her mother, however, had been called to a special teacher conference to discuss the daughter’s deficient work in creativity. It seems the girl had difficulty participating in brainstorming sessions, and was not presenting sufficiently innovative ideas in her projects. As an educator I was dismayed. Why would we be giving a high-performing student a reason to dislike school in the name of creativity? The Aha! insight: far too much attention was being paid to the single role of being the person who “has” the creative idea, rather than the diversity of roles that contribute to the ideas and then integrate them into the group, classroom or society. Trying to teach students to think “outside the box” is an odd demand on education, where the primary mission is to teach the students the details and skills of cultural conventions (literary genres, scientific method, historical controversies and so on – i.e., “boxes”). In contrast, the participatory models fit the mission of education quite well while also promoting the ability to take up creative roles.

Indeed, the participatory models offer several advantages over ideation-focused theories. First, studying how change happens is core to education. Much of that study is in history class, but it can also be important in science, math, art and literature classes. Yes, curricula would have to be revised to emphasize how people pursue creative goals by participating in change through a variety of roles. The fundamental goals of educational courses would not change, however. Second, change is not just a highly salient characteristic of the times in which we live, it is a particularly important interest of young people. And they are already participating in change, taking up formal and informal field functions as they consume products, recommend music or movies or books, transform language and make trends. Few topics could be more directly relevant to their lives. Third, everyone can be involved. Students with lots of unusual ideas are still valued, all the more because of their relations to other students who support, challenge and help them explore and apply new ideas. Students who do not like brainstorming can take up roles that are just as important to the long-term development of ideas as students who do enjoy brainstorming.

Finally, this kind of education prepares students for the world outside of school where a few people become famous for ideas or works, but all have opportunities to participate meaningfully in change. For example, a few artists devote their lives to painting, but many are involved in the art world as critics, gallery owners, collectors, museum curators, and so on. Furthermore, the roles are fluid. Most writers are also readers, and many review books or teach writing. The more students become aware of such roles and how they can fit together for form successful and meaningful lives, the better prepared they may be for the pivots that life often demands over the course of any career.

## Next Steps

If we are going to participate in the ideology of creativity, where do we start? For people in all walks of life, observing and debating the ways we use the concept of creativity will make us more aware of its positive and negative values. Then by applying it the idea of creativity new ways, such as the uses of participatory models in education, we will inevitably gain insight into the values of our complex idea of creativity.

As psychologists, participating in the ongoing evolution of the concept of creativity means developing and applying our own tools: theory, research questions, research methods and applied theory (recommended social practices). Each of these poses particular challenges if advancing the participatory models is our goal.

### Continued Development of Theory

Participatory models have substantial momentum but are still emerging. Among the threads of theory and research that may be usefully woven together are lifelong developmental theories, such as Gruber’s evolving systems (Gruber & Wallace, 1999); distributed cognition theory, which has been applied to creativity by both Sawyer (2010; Sawyer & DeZutter, 2009) and Glăveanu (2014a); and the currently emerging literature on the dangers of creativity (“dark sides,” for example, Cropley, Cropley, Kaufman, & Runco, 2010) and on the ethics of creativity (for example, Moran, Cropley, & Kaufman, 2014).

### Research Questions

Research will develop hand-in-hand with theory. From the participatory perspective, the interesting psychological questions of creativity are not just who has a new idea or how we think of such things. How the initially unthinkable, alien or outlandish become integrated into our concepts of self, group and culture are equally, if not more, salient issues. Who contributes, how they contribute and the variety of roles they take up in the process are just a few of the issues that participatory models add to the mix.

Research on roles is more important to the participatory models than to ideation-focused theories. Here creativity research would borrow from and build on existing social psychology and group dynamics research. The questions of how people have impact through different roles, how they organize multiple roles and how they take up their roles in unexpected ways takes on different significance, though, when viewed as ways of studying creativity.

Positive and negative effects of participating in change also become important. For example, eminence is often used as validation of creativity, but it is a rough and imprecise criterion. The necessarily reductive narratives of history tend to conflate all kinds of major and minor contributors to the genius of a single person. Social, historical and cognitive nuances are lost. But eminence happens. Interesting psychological questions concern its effects. What are the potential effects of organizing one’s identity around goals of becoming famous? Why do some people adapt to fame relatively well, while others do not? Do some people aim at posthumous eminence in their domains but not at current fame – possibly imagining impact through students and protégés, for example? There is some relevant research on these kinds of issues in, for example, historiometric research, social psychology and cultural studies, but, so far, such topics have been far from the mainstream of creativity research.

### Methodologies

In psychology, method and theory always work together. We already have a number of relevant methods, including historical case studies, ethnographic methods and historiometric methods. Part of the methodological challenge may be triangulating among methods more consistently and effectively. Part may also be developing new methods. Dare I say, we have to be *creative* as curators and producers in our discipline? We have a tradition of such creativity, as in Teresa Amabile and associates (1996) demonstrating the statistical reliability and theoretical validity of the commonsense practice of having judges evaluate creative work (consensual assessment technique). Dean Keith Simonton (1984, 2003) has dramatically extended and refined the use of large historical databases to study creativity through historiometrics. More innovative work in methodology will be needed for participatory models and have lasting impact on psychological views of creativity and, as a result, the broader ideology of creativity.

### Applied Technologies

Finally, creativity research has been particularly fertile in producing tools in line with its theories, such as divergent thinking tests, biographical inventories, social trait assessments, idea-generating techniques, problem-solving processes, clinical approaches, inspirational books and curriculum designs. These social practices – psychosocial technologies – bridge the worlds of psychological research and everyday life. Those of us who want to extend participatory models also have to think in these terms. What are the practices that will make these models useful and relevant to people in the many walks of life where the ideology of creativity has impact?

As I wrote earlier, from my own perspective the greatest value of the concept is the question it keeps open: the relationship of individual agency to personal and social change. The participatory models are crucial next steps in that discourse, but there will never be a single answer to the individual-culture question. Hopefully, the participatory models will lead us to deeper understandings of the issues, though, as well as provide practical ways to facilitate and value everyone’s participation in our changing world.
